# Isolated Heme A Synthase from Aquifex aeolicus Is a Trimer

**DOI:** 10.1128/mBio.02615-19

**Published:** 2020-06-30

**Authors:** Hui Zeng, Guoliang Zhu, Shuangbo Zhang, Xinmei Li, Janosch Martin, Nina Morgner, Fei Sun, Guohong Peng, Hao Xie, Hartmut Michel

**Affiliations:** a Department of Molecular Membrane Biology, Max Planck Institute of Biophysics, Frankfurt am Main, Germany; b National Laboratory of Biomacromolecules, Institute of Biophysics, Chinese Academy of Sciences, Beijing, China; c Institute of Physical and Theoretical Chemistry, Goethe University, Frankfurt am Main, Germany; University of California, Irvine

**Keywords:** *Aquifex aeolicus*, cofactor biosynthesis, heme A synthase, hyperthermophilic bacterium, metalloproteins, protein oligomerization, respiratory chain, structural biology

## Abstract

The integral membrane protein heme A synthase (HAS) catalyzes the biosynthesis of heme A, which is a prerequisite for cellular respiration in a wide range of aerobic organisms. Previous studies have revealed that HAS can form homo-oligomeric complexes, and this oligomerization appears to be evolutionarily conserved among prokaryotes and eukaryotes and is shown to be essential for the biological function of eukaryotic HAS. Despite its importance, little is known about the detailed structural properties of HAS oligomers. Here, we aimed to address this critical issue by analyzing the oligomeric state of HAS from Aquifex aeolicus (AaHAS) using a combination of techniques, including size exclusion chromatography coupled with multiangle light scattering (SEC-MALS), cross-linking, laser-induced liquid bead ion desorption mass spectrometry (LILBID-MS), and single-particle electron cryomicroscopy (cryo-EM). Our results show that HAS forms a thermostable trimeric complex. A cryo-EM density map provides information on the oligomerization interface of the AaHAS trimer. These results provide structural insights into HAS multimerization and expand our knowledge of this important enzyme.

## Abstract (precis)

Heme A is a vital redox cofactor unique for the terminal cytochrome *c* oxidase in mitochondria and many microorganisms. It plays a key role in oxygen reduction by serving as an electron carrier and as the oxygen-binding site. Heme A is synthesized from heme O by an integral membrane protein, heme A synthase (HAS). Defects in HAS impair cellular respiration and have been linked to various human diseases, e.g., fatal infantile hypertrophic cardiomyopathy and Leigh syndrome. HAS exists as a stable oligomeric complex, and studies have shown that oligomerization of eukaryotic HAS is necessary for its proper function. However, the molecular architecture of the HAS oligomeric complex has remained uncharacterized. The present study shows that HAS forms trimers and reveals how the oligomeric arrangement contributes to the complex stability and flexibility, enabling HAS to perform its catalytic function effectively. This work provides the basic understanding for future studies on heme A biosynthesis.

## OBSERVATION

Heme A plays an essential role in cellular respiration as an obligatory cofactor in all mitochondrial and many prokaryotic cytochrome *c* oxidases (C*c*O) (1, 2). Heme A synthase (HAS) catalyzes the biosynthesis of heme A from heme O in three sequential steps: (i) a monohydroxylated heme O (heme I) is produced by oxygenation of the C-8 methyl group on pyrrole ring D of heme O; (ii) heme I is further oxidized to a dihydroxylated intermediate; and (iii) spontaneous dehydration of the geminal diol produces the C-8 aldehyde substituent of heme A (2–4). It was shown that the oxygen atom required for this reaction is derived from water (4), even though the HAS activity is strongly dependent on molecular oxygen (3, 5). HAS contains a low-spin heme B; however, its role as a prosthetic group of HAS has not been fully elucidated.

HAS is located in the inner membrane of mitochondria and in the cytoplasmic membrane of prokaryotes. Based on the predicted topology and complement of cysteine residues present in extracellular loops, members of HAS can be classified into four classes (6). Class A is composed of archaeal HAS with only four transmembrane helices (TMHs), while members of classes B, C, and D contain eight TMHs. Classes B and C are found in many bacteria and archaea, which contain one or two pairs of cysteine residues in the extracellular loop region. Class D HAS lacks cysteine residues and represents eukaryotic homologs (Cox15).

HAS from Bacillus subtilis (BsHAS), which belongs to class B, has been relatively well characterized in previous studies (7–11). The functional relevance of several conserved cysteine and histidine residues has been investigated by site-directed mutagenesis (6, 7, 10). However, the complete catalytic mechanism cannot be elucidated due to the lack of an *in vitro* enzyme assay system (2). It has been shown that the purified HAS from Aeropyrum pernix (class A) and B. subtilis may form homo-oligomeric complexes (12). Oligomerization of the eukaryotic HAS has been reported for Cox15 from Saccharomyces cerevisiae (13, 14). Further studies showed that the Cox15 oligomeric complex interacts not only with several C*c*O assembly factors but also with C*c*O subunit I (Cox1), suggesting a critical role in C*c*O assembly and maturation (15, 16). Despite its importance, many questions regarding HAS oligomerization remain unanswered, including the exact oligomeric state and the complex architecture of HAS. Recently, the crystal structure of BsHAS was determined (17). This structure revealed that BsHAS is composed of eight TMHs arranged in two pseudosymmetrically related bundles. However, no information about the oligomeric state could be gained, because BsHAS was crystallized as a monomer.

Therefore, we undertook the present study to examine the oligomeric properties of HAS. The HAS from the hyperthermophilic bacterium Aquifex aeolicus (AaHAS), which belongs to class C, was isolated and studied by comprehensive biochemical, biophysical, and electron-microscopic analyses.

## 

### Purified AaHAS is a *bo*-type cytochrome.

**FIG 1:**
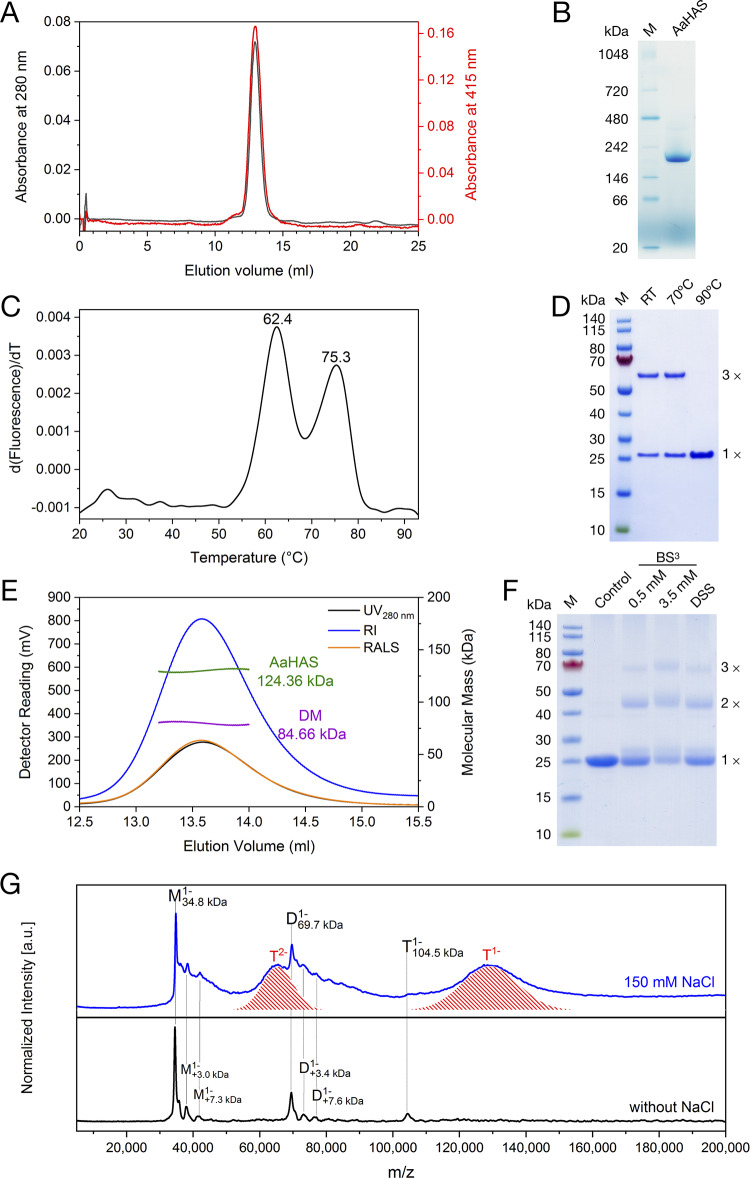
Biochemical and biophysical characterization of AaHAS. (A) Size exclusion profile of the purified AaHAS separated on a Superdex 200 10/300 GL column monitored by recording the absorbance at 280 nm (black) and 415 nm (red). (B) BN-PAGE gel of purified AaHAS. The molecular marker (M) used was the NativeMark unstained protein standard. (C) Thermal denaturation profile of AaHAS analyzed by nano-DSF. The corresponding transition temperatures are indicated. (D) SDS-PAGE analyses of AaHAS. Purified AaHAS was incubated with 10 mM dithiothreitol (DTT) at room temperature (RT), 70°C, and 90°C for 10 min prior to SDS-PAGE. The molecular marker (M) used was the PageRule prestained protein ladder. (E) SEC-MALS analysis of purified AaHAS. The readings of UV, refractive index (RI), and light scattering (RALS) detectors are shown in black, blue, and orange, respectively. The purple line indicates the contribution of the detergent DM, and the green line shows the calculated molecular mass of the AaHAS trimeric complex. (F) Cross-linking analyses of AaHAS. Purified AaHAS was subjected to 0.5/3.5 mM BS^3^ or 0.5 mM DSS cross-linking at room temperature for 30 min. After quenching with 50 mM Tris-HCl (pH 7.5), samples were incubated at 90°C for 10 min and subjected to SDS-PAGE. (G) Identification of the trimeric AaHAS complex by LILBID-MS. LILBID spectra of purified AaHAS in the presence of 150 mM NaCl (top) and without NaCl (bottom) are shown. The red shadings in the upper spectrum represent the trimeric AaHAS with tightly bound lipids. The positions of the AaHAS monomer (M), dimer (D), and trimer (T) are indicated.

AaHAS was heterologously expressed in Escherichia coli and purified to high homogeneity using affinity chromatography. From size exclusion chromatography, the purified AaHAS eluted as a monodisperse peak which was monitored at both 280 and 415 nm, indicating the incorporation of heme cofactors (Fig. 1A). To characterize the heme cofactors, absorption spectra of as-isolated, fully oxidized, and fully reduced AaHAS were recorded (see Fig. S1 in the supplemental material). Optical absorption spectra of as-isolated and fully reduced AaHAS were almost identical. In the reduced state, AaHAS has an absorption peak at 427 nm in the Soret region, a β-peak at 528 nm, and a broad α-absorption at 560 nm, indicating the perturbation of the low-spin heme B and probably of heme O. After oxidation with potassium ferricyanide, the Soret peak shifted to 415 nm and decreased in intensity. In addition, spectral properties of purified AaHAS are similar to those observed for BsHAS (9), with the exception that no absorption peak was observed at about 585 nm in the reduced-minus-oxidized difference spectrum, suggesting the absence of heme A in the purified AaHAS preparation. To determine whether heme O is incorporated, AaHAS was analyzed by high-performance liquid chromatography with UV detection coupled to mass spectrometry (Fig. S2). Both heme O and heme B, but not heme A, were identified in the purified AaHAS, which is consistent with our spectroscopic data. In addition, pyridine hemochrome assays were performed (Fig. S3), and a stoichiometry of 1.1 hemes (heme B+O) to 1 AaHAS monomer was found.

10.1128/mBio.02615-19.1 FIG S1 UV-visible spectra (UV-vis) of AaHAS. (A) Absorption spectra of as-isolated (black), fully reduced (red), and fully oxidized (blue) AaHAS (6 μM) were recorded with a Lambda 35 UV-vis spectrometer. The inset enlarges the 500- to 600-nm region. (B) Reduced-minus-oxidized difference spectrum of AaHAS. Download FIG S1, TIF file, 0.1 MB. Copyright © 2020 Zeng et al. 2020 Zeng et al. This content is distributed under the terms of the Creative Commons Attribution 4.0 International license.

10.1128/mBio.02615-19.2 FIG S2 Identification of the heme cofactors in AaHAS using high-performance liquid chromatography with UV detection coupled to mass spectrometry (HPLC-UV-MS). HPLC-UV-MS experiments for on-column extraction and heme analysis were carried out on an Ultimate 3000 RSLC system (Dionex) equipped with a CSH C_18_ column (Waters) and VWD detector set to 400 nm, which was directly coupled to an Impact II mass spectrometer (Bruker Daltonik). Loading, extraction, and separation were carried out using water (A) and acetonitrile-water (95:5 [vol/vol]) (B), both supplemented with 0.1% formic acid, as mobile phases with a flow rate of 300 μl/min at 55°C. After 2 min of loading with 2% B, a linear gradient was ramped from 2% to 95% B in 28 min followed by a 5-min wash (95% B) and 5-min equilibration (2% B). The mass spectrometer was operated in positive ion mode with a mass range from *m/z* 50 to 1,300. Heme identification was conducted through correlation of accurate mass extracted-ion chromatograms, UV absorption, and matching Fe isotope patterns. The positions where heme O and heme B elute are indicated. Download FIG S2, TIF file, 0.7 MB. Copyright © 2020 Zeng et al. 2020 Zeng et al. This content is distributed under the terms of the Creative Commons Attribution 4.0 International license.

10.1128/mBio.02615-19.3 FIG S3 Pyridine hemochrome spectra for hemes isolated from AaHAS. The pyridine hemochrome assay was carried out as described by E. A. Berry and B. L. Trumpower (Anal Biochem 161:1–15, 1987). Pyridine hemochrome spectra (500 to 600 nm) of oxidized and reduced hemes isolated from AaHAS are shown in black and red, respectively. Using an extinction coefficient ε_(R-O, 557-540)_ of 23.98 mM^−1^ cm^−1^ for pyridine hemochrome *b*, the heme content was determined to be 315 ± 14 μM (data are means ± SD from three experiments). Because heme O can be also identified in the purified HAS and the pyridine hemochrome spectrum of heme O is similar to that of heme B, this value may represent the total content of hemes (heme B+O) rather than heme B alone. The concentration of AaHAS was determined to be 284 ± 5 μM (data are means ± standard deviations from three experiments) using quantitative amino acid analysis at the Functional Genomics Center Zurich. Download FIG S3, TIF file, 0.1 MB. Copyright © 2020 Zeng et al. 2020 Zeng et al. This content is distributed under the terms of the Creative Commons Attribution 4.0 International license.

### AaHAS forms a thermostable trimeric complex.

The protein sample was subjected to N-terminal sequencing, and the results showed that the initiator methionine has been cleaved from the mature AaHAS. Therefore, the expected molecular mass of the monomeric apo-AaHAS is 34.96 kDa, considering the size of the affinity tag Strep-Tag II. To determine the oligomeric state of AaHAS, blue native polyacrylamide gel electrophoresis (BN-PAGE) analysis was conducted, and a single dominant band migrating at approximately 200 kDa was detected (Fig. 1B). This result is similar to that found in previous studies (13, 18), where COX15 from S. cerevisiae exists as an oligomeric complex as observed on BN-PAGE analysis. Since the migration behavior of membrane proteins in a BN-PAGE gel is strongly influenced by the bound detergents and Coomassie dye molecules, a conversion factor of 1.8 was used (19), yielding a mass of 110 kDa after correction, which is very close to what is expected for a trimer (105 kDa).

We analyzed the purified AaHAS by sodium dodecyl sulfate (SDS)-PAGE. Two distinct bands with apparent molecular masses of about 25 and 70 kDa were visible (Fig. 1D). To correlate the observed masses with different oligomeric states, a matrix-assisted laser desorption ionization time-of-flight mass-spectrometric analysis was conducted (data not shown). The actual sizes of the polypeptides present in the lower and upper bands were determined to be 34.95 kDa and 104.56 kDa, corresponding to the masses of the monomeric and trimeric forms of AaHAS, respectively. The purified AaHAS was also subjected to heat treatment prior to SDS-PAGE. The trimeric band remained largely unaffected after exposure to temperatures up to 70°C but disappeared completely when the synthase was heated to 90°C (Fig. 1D). It should be noted that the optimal growth temperature for *A. aeolicus* is 85°C. This unusually high thermal stability of the AaHAS trimer was further confirmed by nano-differential scanning fluorimetry, which showed two melting temperatures, 62.4°C and 75.3°C, probably due to the presence of two distinct thermally induced transitions (Fig. 1C). Interestingly, it was shown that HAS from the hyperthermophilic archaeon *A. pernix* is also very heat stable (12). In addition, chemical cross-linking experiments were performed using amine-reactive cross-linkers. Despite the heat treatment, when AaHAS was subjected to bissulfosuccinimidyl suberate (BS^3^) and disuccinimidyl suberate (DSS) cross-linking and subsequently analyzed by SDS-PAGE, the accumulation of dimeric and trimeric forms became clearly detectable (Fig. 1F).

To investigate the oligomeric state of AaHAS in detergent solution, we performed size exclusion chromatography-multiangle light scattering (SEC-MALS) experiments. The averaged masses of the AaHAS complex with and without the bound detergents were determined to be 209.02 kDa and 124.36 kDa (Fig. 1E). The latter value is slightly higher than expected but is still in agreement with the predicted molecular mass of the trimeric AaHAS with bound cofactors and lipids. In addition to SEC-MALS, laser-induced liquid bead ion desorption mass spectrometry (LILBID-MS) was also used. Under the harsh-laser condition, the LILBID-MS spectrum showed a dominating trimer of AaHAS, which is observed as a broad peak ranging from *m*/*z* 100,000 to 150,000 and as a doubly negatively charged species centered at 65,000 (Fig. 1G, top). The broadened feature is due to the mass of additional lipids, which are not removed in the LILBID process, suggesting that these lipids are tightly bound to the AaHAS, probably at the oligomerization interface. In addition, LILBID-MS measurements were also performed on additionally desalted protein samples. As shown in Fig. 1G (bottom), mainly mono- and dimeric AaHAS species were observed, as indicated by two prominent *m*/*z* signals of 34,500 and 69,500. This result can be attributed to the decreased stability of AaHAS trimer when desalted. Nevertheless, trimeric AaHAS is still detectable as a singly negatively charged peak at *m*/*z* 105,500. Taken together, our LILBID-MS analyses showed that lipids may play an important role in the stabilization and assembly of the trimeric AaHAS complex.

### The AaHAS trimer is mainly formed by hydrophobic interactions.

**FIG 2:**
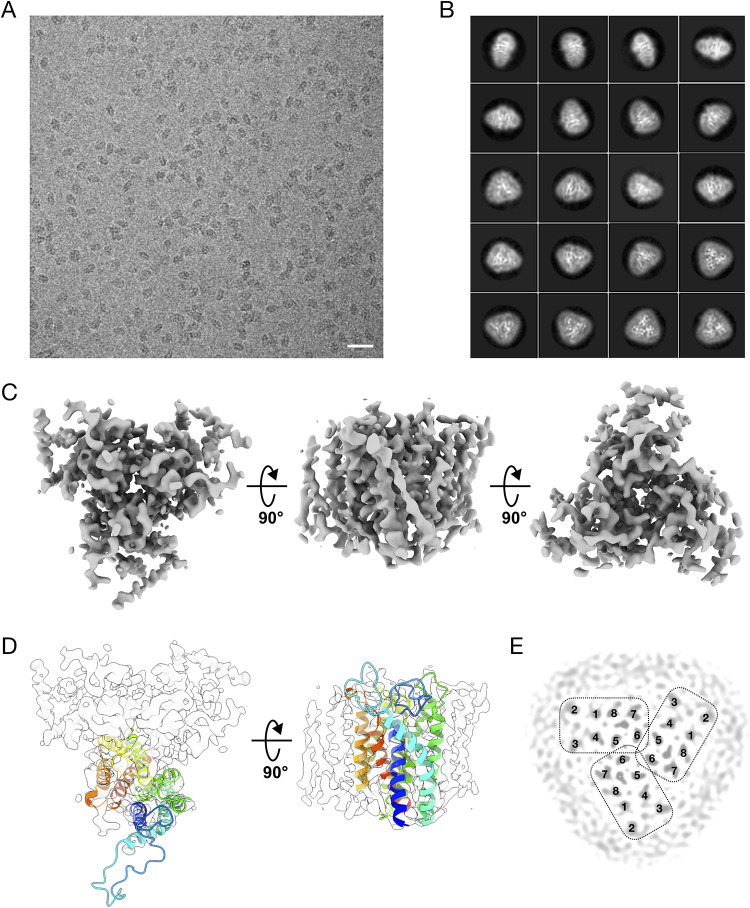
Structure of the AaHAS trimer determined by 3D reconstruction of single-particle cryo-EM images. (A) Typical cryo-EM micrograph of AaHAS imaged with a FEI Titan Krios and a Gatan K2 Summit camera. Bar, 200 Å. (B) Selection of 20 reference-free 2D class averages of AaHAS showing secondary structures in projection. (C) Surface-rendered views of the final 3D map. (D) Fitting of the homology model of AaHAS into the cryo-EM density map by rigid-body fitting in UCSF Chimera. The AaHAS model, which was built using the X-ray crystallographic coordinates of the HAS from B. subtilis (PDB entry 6A2J) as a template, is shown as cartoon representation and colored using a rainbow gradient from the N terminus (blue) to the C terminus (red). (E) Cross-section of the map, illustrating the arrangements of the transmembrane helices in the AaHAS trimer. The transmembrane helices of each AaHAS monomer are numbered 1 to 8.

To gain more insight into the structure of the AaHAS homo-oligomer complex, we used single-particle electron cryomicroscopy (Fig. 2A). A total of ∼52,000 particles were selected from 528 micrographs, and the reference-free two-dimensional (2D) class averages clearly showed triangle-shaped top views and rod-shaped side views of AaHAS complex (Fig. 2B). Three-dimensional (3D) reconstruction and further refinement were performed using RELION (20), resulting in an AaHAS reconstruction at 4.6 Å resolution without applied symmetry (data not shown) and the final map at 4.2 Å resolution with applied C3 symmetry (Fig. 2C). At a low-density threshold, the map shows that a disordered belt of detergents and lipids surrounds the hydrophobic domain of AaHAS complex and defines the position of the membrane (Fig. S5). Twenty-four transmembrane helices that can be grouped into three independent domains are clearly observed at intermediate density threshold, which leaves no doubt that AaHAS assembles as a trimer. Due to the lack of density in the loops between transmembrane helices, an unambiguous structural model cannot be constructed. Therefore, a homology model of AaHAS was built based on the crystal structure of BsHAS using the Swiss-Model server (21). Fitting of the resulting model into the cryo-EM map showed that, except for the loop region between TM1 and TM2, the majority of the AaHAS model matched relatively well with the experimental map (Fig. 2D). Three AaHAS protomers are arranged in a propeller-shaped manner surrounding the 3-fold symmetr*y* axis. A cross-section view of the AaHAS map revealed that the C bundles (transmembrane region 5 [TM5] to TM8) of three AaHAS monomers are tightly associated with each other, forming the core of the trimeric complex (Fig. 2E). The central trimer interface is mainly constituted by TM5 and TM6 through hydrophobic interactions. Furthermore, extra density was found only in the C bundle, which can be attributed to the presence of heme B or heme O. The recently published crystal structure of BsHAS has revealed that heme B is present in the C bundle and the N bundle is the substrate-heme (heme O) binding domain (17). Therefore, the core of the trimeric complex is expected to be fairly rigid, since heme B is embedded in the C bundle of AaHAS via coordination of its heme iron by two histidine residues. On the other hand, the N bundles (TM1 to TM4) of AaHAS constitute the peripheral domain of the trimeric complex. The peripheral region is likely to be flexible, because the N bundle of AaHAS may undergo conformational changes to allow the uptake of the substrate heme O and the release of the reaction product heme A.

### Conclusion.

In this study, we investigated the oligomeric state of AaHAS using complementary methods. Our results explicitly indicate that AaHAS exists mainly as a trimer. We provide a structural basis for understanding how HAS forms stable oligomeric complexes. Our results might serve as a useful guide for further studies on the structure and function of HAS.

### Methods.

**(i) Construction of expression vector.** Genomic DNA from Aquifex aeolicus strain VF5 was isolated using the G-spin genomic DNA extraction kit (iNtRON Biotechnology). The coding sequence of the heme A synthase gene (*ctaA*; gene ID, 1192686) was amplified using the primer pair *ctaA*-F (5′-CACGGATCCATGAACACCAACCTC) and *ctaA*-R (5′-GTGGAATTCTGCCAGCTCTCCTC), and the resulting 906-bp DNA fragment was cloned into the pJET1.2 vector (Thermo Fisher Scientific). The resulting plasmid, pJET1.2-*ctaA*, was digested with NcoI and EcoRI and subsequently ligated into the corresponding sites of pBADC3 (22), which contains a C-terminal Strep-Tag II, yielding the expression vector pBADC3-*ctaA*.

**(ii) Protein production and purification.** For heterologous overexpression, Escherichia coli Top10 cells transformed with pBADC3-*ctaA* vector were grown at 32°C in lysogeny broth (LB) medium supplemented with 100 μg/ml ampicillin to an optical density at 600 nm (OD_600_) of 0.5. Production of heme A synthase was induced by addition of 0.02% (wt/vol) l-arabinose, and incubation was continued for 4 h. Cells were harvested by centrifugation and subsequently resuspended in lysis buffer (20 mM Tris-HCl [pH 7.4], 150 mM NaCl, 1 mM phenylmethylsulfonylfluoride [PMSF], 20 μg/ml DNase I]. Cells were disrupted by passing them through a French pressure cell at 12,000 lb/in^2^ for 3 cycles. The cell lysate was centrifuged at 4°C and 10,000 × *g* for 1 h. The supernatant containing the membranes was centrifuged at 4°C and 200,000 × *g* for 3 h. The pelleted membranes were resuspended in membrane resuspension buffer (20 mM Tris-HCl [pH 7.4], 150 mM NaCl), and membrane proteins were solubilized by slow stirring with 1% (wt/vol) *n*-decyl-β-d-maltoside (DM) at 4°C for 1 h. The insoluble membrane fraction was removed by ultracentrifugation at 200,000 × *g* for 1 h, and the supernatant containing the solubilized AaHAS was supplemented with avidin (0.05 mg avidin per mg of total solubilized protein) and subsequently incubated with Strep-Tactin resin (IBA) pre-equilibrated with purification buffer (20 mM Tris-HCl [pH 7.4], 150 mM NaCl, 0.15% [wt/vol] DM) at 4°C for 1 h. After incubation, the resin was washed with 15 column volumes (CV) of purification buffer. Strep-Tag II-tagged AaHAS was eluted with 5 CV of the purification buffer supplemented with 5 mM desthiobiotin. The protein was purified further on a Superdex 200 10/300 GL column (GE Healthcare) equilibrated with purification buffer. Peak fractions containing AaHAS were collected, concentrated, and stored at −80°C. The total protein concentration was determined using the bicinchoninic acid (BCA) assay (Pierce). Quantitative amino acid analysis was used to accurately determine the concentration of purified AaHAS at the Functional Genomics Center Zurich. An extinction coefficient of 192 mM^−1 ^cm^−1^ was determined for the reduced AaHAS at 427 nm.

**(iii) SDS-PAGE, BN-PAGE, and chemical cross-linking.** Sodium dodecyl sulfate-polyacrylamide gel electrophoresis (SDS-PAGE) was performed using 4 to 12% bis-Tris NuPAGE gels (Invitrogen) followed by Coomassie blue staining. Blue native-PAGE was performed using Novex 4 to 16% bis-Tris gels (Invitrogen) according to the manufacturer’s instructions. Chemical cross-linking experiments using bissulfosuccinimidyl suberate (BS^3^) or disuccinimidyl suberate (DSS) were performed according to the manufacturer’s instructions (Thermo Fisher Scientific). The reaction was performed at room temperature for 30 min and quenched by dilution with 50 mM Tris-HCl (pH 7.5).

**(iv) Nano-differential scanning fluorimetry.** Nano-differential scanning fluorimetry (nano-DSF) was performed by using a Prometheus NT.48 nano-DSF instrument (NanoTemper Technologies). Measurements were performed at an AaHAS concentration of 1 mg/ml using the following settings: 15 to 98°C, 0.5°C/min.

**(v) Size exclusion chromatography coupled with multiangle light scattering.** SEC-MALS experiments were performed using a Viscotek SEC-MALS 20 system (Malvern) at 4°C. Samples (150 μl AaHAS at ∼2 mg/ml) were loaded onto a Superdex 200 10/300 GL column pre-equilibrated with purification buffer. Data were analyzed using OmniSEC software (Malvern). The specific refractive index increment (*dn*/*dc*) of the protein was 0.187 ml/g. The *dn*/*dc* value of DM was taken as 0.12 ml/g (23). The molar extinction coefficient of AaHAS at 280 nm was calculated to be 50,435 M^−1 ^cm^−1^ using the ProtParam server.

**(vi) Laser-induced liquid bead ion desorption mass spectrometry.** Prior to LILBID-MS, AaHAS (2 mg/ml) samples were buffer exchanged into 20 mM Tris-HCl (pH 7.4), 0.15% DM. Release of solvated AaHAS ions from solution was achieved by irradiating microdroplets (diameter, ∼50 μm; volume, ∼65 pl) with pulsed infrared laser light (λ = 3 μm) (24). The laser was a standard Nd:YAG laser and worked with a maximum energy of 23 mJ (harsh mode). Ion optics based on a Wiley-McLaren type accelerator are used to transfer the ions into a homebuilt time-of-flight (TOF) setup for mass analysis. More detailed information regarding LILBID was published previously (25). Ion detection was performed in negative-ion mode.

**(vii) Single-particle cryo-electron microscopy.** To prepare the cryo-electron microscopy (cryo-EM) grids, 3 μl of the purified AaHAS (2.5 mg/ml) was applied to glow-discharged holey carbon film grids (GIG Au R 1/1, 300 mesh), which were plasma cleaned for 1 min using the Gatan Solarus system. Grids were blotted with Whatman 595 filter paper for 3 s at 16°C and 100% humidity and then flash frozen in liquid ethane using a Vitrobot (FEI). Grids were loaded onto a FEI Titan Krios transmission electron microscope operated at 300 kV. Images were collected using SerialEM (26) software on a K2 detector in the superresolution counting mode at a calibrated magnification of ×165,000 (yielding a pixel size of 0.41 Å) and a dose rate of 8.45 e^−^/Å^2^/s. A Quantum energy filter (Gatan), whose energy slot was set to 20 eV, was applied to remove any inelastic scattering. Defocus value varied from −1.5 to −2.5 μm. During movie data collection, frame alignment, exposure weighting, and contrast transfer function parameter estimation were performed using the cryo-EM platform with automated data collection scripts integrated with Motioncor2 (27) and CTFFIND4 (28) at the Institute of Biophysics of the Chinese Academy of Sciences. Particle picking was performed using Gautomatch (https://www2.mrc-lmb.cam.ac.uk/research/locally-developed-software/zhang-software/) without a template, and particle analysis and 3D reconstruction were performed using RELION (20). Detailed information about data collection and processing is provided in Fig. S4 and Table S1.

10.1128/mBio.02615-19.4 FIG S4 Cryo-EM analysis of AaHAS. (A) Cryo-EM data processing workflow of AaHAS. Gautomatch was used to pick 103,088 particles without template from 528 micrographs. They were extracted and sorted by two rounds of 2D classification. Approximately 13,000 particles were selected to generate the initial mode. Then, 52,065 particles were re-extracted and used for 3D autorefinement and postprocessing, yielding a map at 4.5 Å resolution. This was subjected to CTF refinement and further postprocessing, yielding a final map at 4.2 Å resolution. (B) Fourier shell correlation (FSC) curve for the 3D reconstruction of the AaHAS cryo-EM map. The average resolution is estimated to be 4.2 Å on the basis of the FSC value of 0.143. (C) Local resolution estimation of the cryo-EM map. (D) Euler angle distribution of particles used in the final reconstruction. Download FIG S4, TIF file, 2.0 MB. Copyright © 2020 Zeng et al. 2020 Zeng et al. This content is distributed under the terms of the Creative Commons Attribution 4.0 International license.

10.1128/mBio.02615-19.5 FIG S5 3D cryo-EM density map of AaHAS trimer. Top view (left) and side view (right) of the density map. At a low-density threshold, the map shows that a belt of disordered detergents and lipids (light grey) surrounds the AaHAS complex (dark blue). Download FIG S5, TIF file, 1.9 MB. Copyright © 2020 Zeng et al. 2020 Zeng et al. This content is distributed under the terms of the Creative Commons Attribution 4.0 International license.

10.1128/mBio.02615-19.6 TABLE S1 Statistics of cryo-EM data collection and reconstruction. Download Table S1, DOCX file, 0.01 MB. Copyright © 2020 Zeng et al. 2020 Zeng et al. This content is distributed under the terms of the Creative Commons Attribution 4.0 International license.

### Data availability.

The EM map was deposited in EMDB with the accession number EMD-10987.

## References

[1] Kim HJ, Khalimonchuk O, Smith PM, Winge DR 2012 Structure, function, and assembly of heme centers in mitochondrial respiratory complexes. Biochim Biophys Acta 1823:1604–1616. doi:10.1016/j.bbamcr.2012.04.008. 22554985 PMC3601904

[2] Hederstedt L 2012 Heme A biosynthesis. Biochim Biophys Acta 1817:920–927. doi:10.1016/j.bbabio.2012.03.025. 22484221

[3] Brown KR, Allan BM, Do P, Hegg EL 2002 Identification of novel hemes generated by heme A synthase: evidence for two successive monooxygenase reactions. Biochemistry 41:10906–10913. doi:10.1021/bi0203536. 12206660

[4] Brown KR, Brown BM, Hoagland E, Mayne CL, Hegg EL 2004 Heme A synthase does not incorporate molecular oxygen into the formyl group of heme A. Biochemistry 43:8616–8624. doi:10.1021/bi049056m. 15236569

[5] Hannappel A, Bundschuh FA, Ludwig B 2011 Characterization of heme-binding properties of Paracoccus denitrificans Surf1 proteins. FEBS J 278:1769–1778. doi:10.1111/j.1742-4658.2011.08101.x. 21418525

[6] Lewin A, Hederstedt L 2016 Heme A synthase in bacteria depends on one pair of cysteinyls for activity. Biochim Biophys Acta 1857:160–168. doi:10.1016/j.bbabio.2015.11.008. 26592143

[7] Hederstedt L, Lewin A, Throne-Holst M 2005 Heme A synthase enzyme functions dissected by mutagenesis of Bacillus subtilis CtaA. J Bacteriol 187:8361–8369. doi:10.1128/JB.187.24.8361-8369.2005. 16321940 PMC1317025

[8] Lewin A, Hederstedt L 2008 Promoted evolution of a shortened variant of heme A synthase in the membrane of Bacillus subtilis. FEBS Lett 582:1330–1334. doi:10.1016/j.febslet.2008.03.015. 18358840

[9] Svensson B, Hederstedt L 1994 Bacillus subtilis CtaA is a heme-containing membrane protein involved in heme A biosynthesis. J Bacteriol 176:6663–6671. doi:10.1128/JB.176.21.6663-6671.1994. 7961419 PMC197023

[10] Mogi T 2009 Probing structure of heme A synthase from Bacillus subtilis by site-directed mutagenesis. J Biochem 145:625–633. doi:10.1093/jb/mvp017. 19174544

[11] Svensson B, Andersson KK, Hederstedt L 1996 Low-spin heme A in the heme A biosynthetic protein CtaA from Bacillus subtilis. Eur J Biochem 238:287–295. doi:10.1111/j.1432-1033.1996.0287q.x. 8665949

[12] Lewin A, Hederstedt L 2006 Compact archaeal variant of heme A synthase. FEBS Lett 580:5351–5356. doi:10.1016/j.febslet.2006.08.080. 16989823

[13] Swenson S, Cannon A, Harris NJ, Taylor NG, Fox JL, Khalimonchuk O 2016 Analysis of oligomerization properties of heme a synthase provides insights into its function in eukaryotes. J Biol Chem 291:10411–10425. doi:10.1074/jbc.M115.707539. 26940873 PMC4858986

[14] Khalimonchuk O, Kim H, Watts T, Perez-Martinez X, Winge DR 2012 Oligomerization of heme o synthase in cytochrome oxidase biogenesis is mediated by cytochrome oxidase assembly factor Coa2. J Biol Chem 287:26715–26726. doi:10.1074/jbc.M112.377200. 22669974 PMC3411010

[15] Bareth B, Dennerlein S, Mick DU, Nikolov M, Urlaub H, Rehling P 2013 The heme a synthase Cox15 associates with cytochrome c oxidase assembly intermediates during Cox1 maturation. Mol Cell Biol 33:4128–4137. doi:10.1128/MCB.00747-13. 23979592 PMC3811676

[16] Taylor NG, Swenson S, Harris NJ, Germany EM, Fox JL, Khalimonchuk O 2017 The assembly factor Pet117 couples heme a synthase activity to cytochrome oxidase assembly. J Biol Chem 292:1815–1825. doi:10.1074/jbc.M116.766980. 27998984 PMC5290955

[17] Niwa S, Takeda K, Kosugi M, Tsutsumi E, Mogi T, Miki K 2018 Crystal structure of heme A synthase from Bacillus subtilis. Proc Natl Acad Sci U S A 115:11953–11957. doi:10.1073/pnas.1813346115. 30397130 PMC6255202

[18] Herwaldt EJ, Rivett ED, White AJ, Hegg EL 2018 Cox15 interacts with the cytochrome bc1 dimer within respiratory supercomplexes as well as in the absence of cytochrome c oxidase. J Biol Chem 293:16426–16439. doi:10.1074/jbc.RA118.002496. 30181213 PMC6200925

[19] Heuberger E, Veenhoff LM, Duurkens RH, Friesen RHE, Poolman B 2002 Oligomeric state of membrane transport proteins analyzed with blue native electrophoresis and analytical ultracentrifugation. J Mol Biol 317:591–600. doi:10.1006/jmbi.2002.5416. 11955011

[20] Scheres S 2012 RELION: implementation of a Bayesian approach to cryo-EM structure determination. J Struct Biol 180:519–530. doi:10.1016/j.jsb.2012.09.006. 23000701 PMC3690530

[21] Schwede T, Kopp J, Guex N, Peitsch MC 2003 SWISS-MODEL: an automated protein homology-modeling server. Nucleic Acids Res 31:3381–3385. doi:10.1093/nar/gkg520. 12824332 PMC168927

[22] Surade S, Klein M, Stolt BP, Muenke C, Roy A, Michel H 2006 Comparative analysis and “expression space” coverage of the production of prokaryotic membrane proteins for structural genomics. Protein Sci 15:2178–2189. doi:10.1110/ps.062312706. 16943447 PMC2242615

[23] Lopez-Redondo ML, Coudray N, Zhang Z, Alexopoulos J, Stokes DL 2018 Structural basis for the alternating access mechanism of the cation diffusion facilitator YiiP. Proc Natl Acad Sci U S A 115:3042–3047. doi:10.1073/pnas.1715051115. 29507252 PMC5866550

[24] Morgner N, Kleinschroth T, Barth H-D, Ludwig B, Brutschy B 2007 A novel approach to analyze membrane proteins by laser mass spectrometry: from protein subunits to the integral complex. J Am Soc Mass Spectrom 18:1429–1438. doi:10.1016/j.jasms.2007.04.013. 17544294

[25] Peetz O, Hellwig N, Henrich E, Mezhyrova J, Dötsch V, Bernhard F, Morgner N 2019 LILBID and nESI: different native mass spectrometry techniques as tools in structural biology. J Am Soc Mass Spectrom 30:181–191. doi:10.1007/s13361-018-2061-4. 30225732 PMC6318263

[26] Mastronarde DN 2005 Automated electron microscope tomography using robust prediction of specimen movements. J Struct Biol 152:36–51. doi:10.1016/j.jsb.2005.07.007. 16182563

[27] Zheng SQ, Palovcak E, Armache J-P, Verba KA, Cheng Y, Agard DA 2017 MotionCor2: anisotropic correction of beam-induced motion for improved cryo-electron microscopy. Nat Methods 14:331–332. doi:10.1038/nmeth.4193. 28250466 PMC5494038

[28] Rohou A, Grigorieff N 2015 CTFFIND4: fast and accurate defocus estimation from electron micrographs. J Struct Biol 192:216–221. doi:10.1016/j.jsb.2015.08.008. 26278980 PMC6760662

