# Supplementary figures and images for: Isolated Heme A Synthase from Aquifex aeolicus Is a Trimer
Source: mBio. 2020 Jun 30;11(3):e02615-19. doi: 10.1128/mBio.02615-19 (PMC7327177; doi:10.1128/mBio.02615-19)

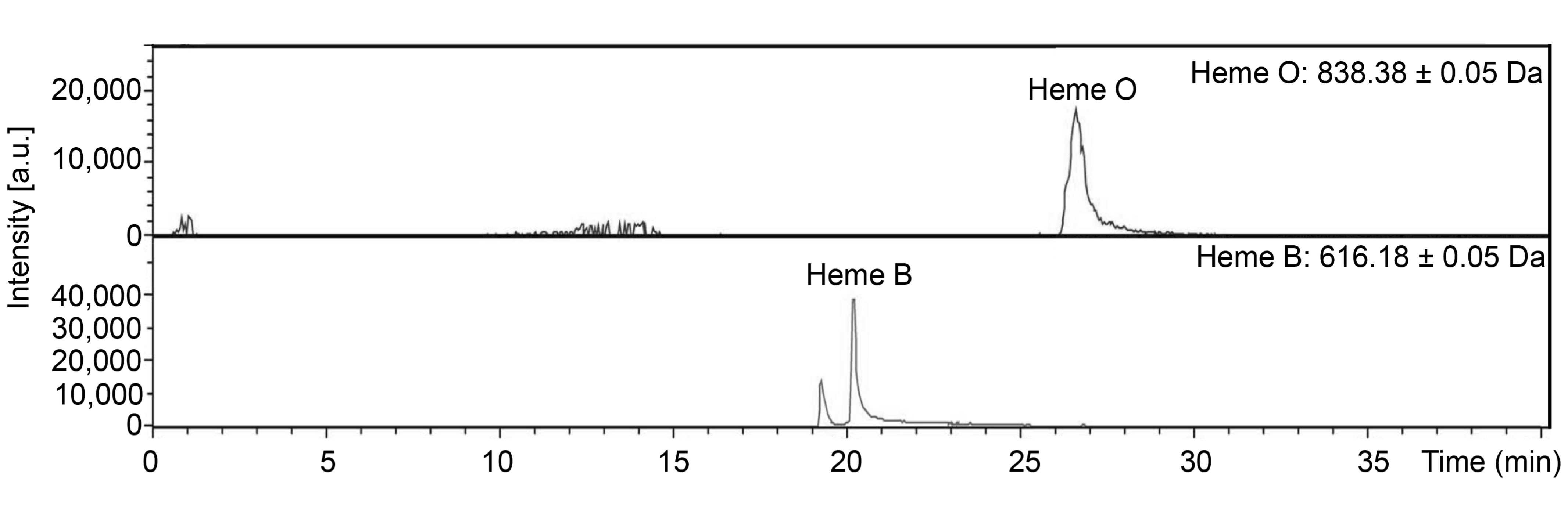

Supplement: FIG S2 [file mBio.02615-19-sf002.tif]

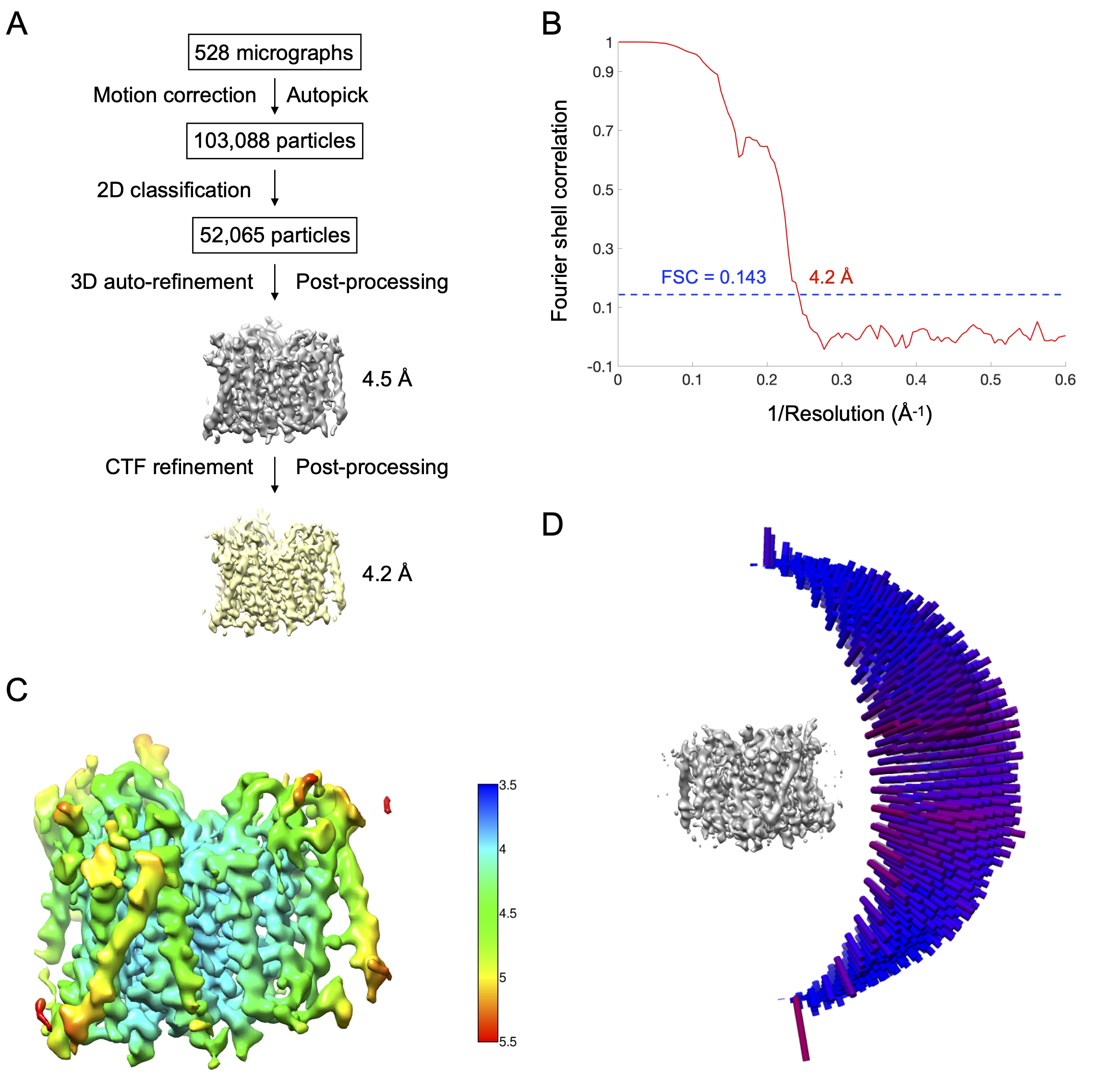

Supplement: FIG S4 [file mBio.02615-19-sf004.tif]

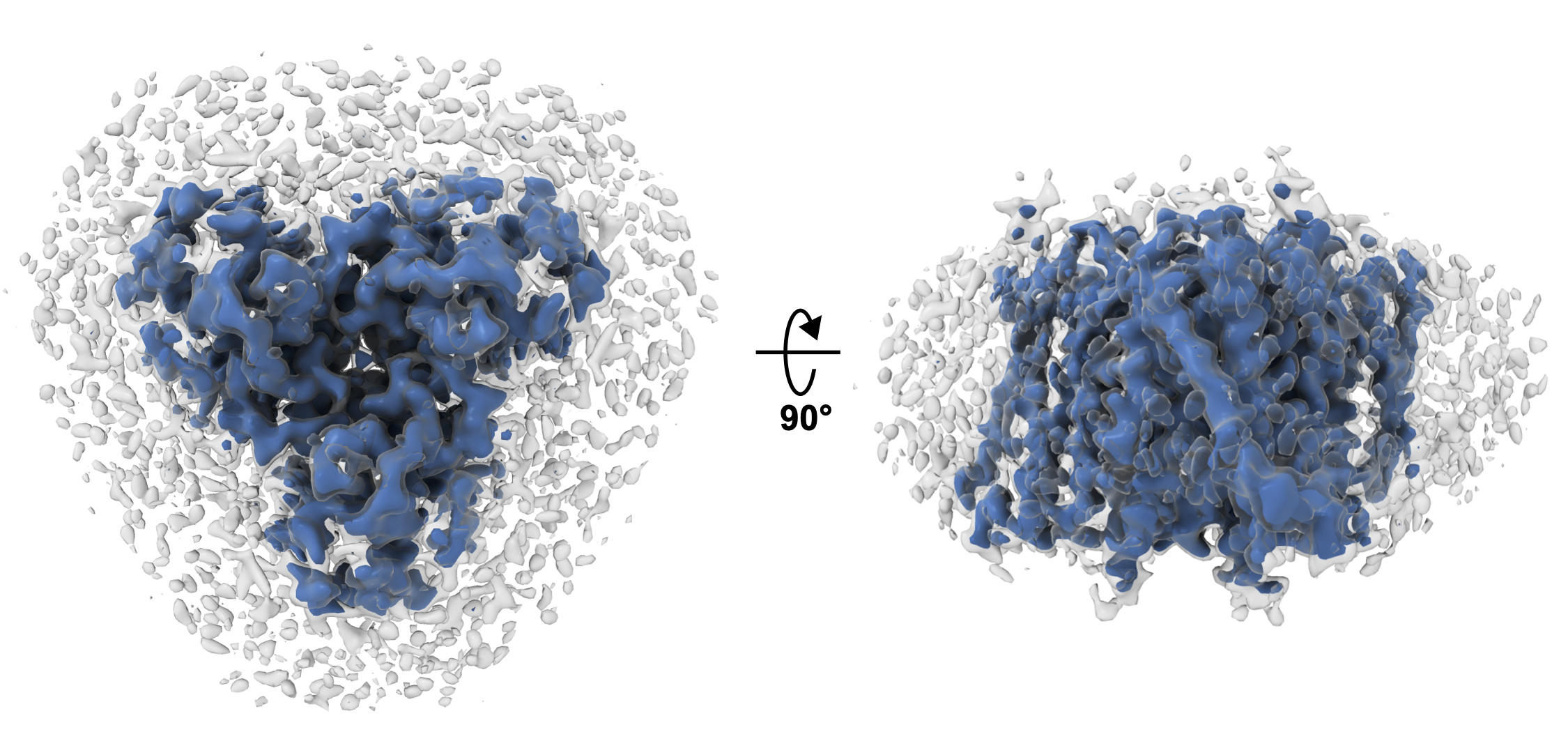

Supplement: FIG S5 [file mBio.02615-19-sf005.tif]
