# Supplementary material for: Isolated Heme A Synthase from Aquifex aeolicus Is a Trimer
Source: mBio. 2020 Jun 30;11(3):e02615-19. doi: 10.1128/mBio.02615-19 (PMC7327177; doi:10.1128/mBio.02615-19)
Supplement: TABLE S1 [file mBio.02615-19-st001.docx]

Table S1: Statistics of cryo-EM data collection and reconstruction

|  | **AaHAS (EMD-10987)** |
| --- | --- |
| **Data collection** | |
| **Microscope** | FEI Titan Krios |
| **Voltage (kV)** | 300 |
| **Detector** | Gatan Bioquantum K2 |
| **Energy filter** | 20 eV |
| **Pixel size (Å/pixel)** | 0.41 |
| **Electron dose (e/Å^2^)** | 60 |
| **Defocus range (μm)** | -1.5~-2.5 |
| **Reconstruction** | |
| **Software** | RELION 3.0-beta / RELION 2.0 |
| **Micrographs** | 528 |
| **Particles selected** | 52,065 |
| **Accuracy of rotation** | 2.768 |
| **Accuracy of translations (pixel)** | 1.253 |
| **Symmetry** | C3 |
| **Map sharpening B-factor (Å^2^)** | -225.4  4.2 |
| **Final resolution (Å)** |  |
